# Ambient dose equivalent rates of gamma radiation from natural radionuclides and ^137^Cs at grasslands and forests in the area of the Belarusian NPP in the pre-commissioning period (2019)

**DOI:** 10.1093/rpd/ncae016

**Published:** 2024-02-13

**Authors:** Valery Ramzaev, Christian Bernhardsson, Aleksandr Vodovatov, Larisa Chipiga, Vladislav Nekrasov, Alexander Dvornik

**Affiliations:** Saint-Petersburg Research Institute of Radiation Hygiene after Professor P.V. Ramzaev, 8 Mira Str., Saint-Petersburg, 197101, Russia; Medical Radiation Physics, Department of Translational Medicine, Lund University, SE-205 02 Malmö, Sweden; Saint-Petersburg Research Institute of Radiation Hygiene after Professor P.V. Ramzaev, 8 Mira Str., Saint-Petersburg, 197101, Russia; Saint-Petersburg Research Institute of Radiation Hygiene after Professor P.V. Ramzaev, 8 Mira Str., Saint-Petersburg, 197101, Russia; Saint-Petersburg Research Institute of Radiation Hygiene after Professor P.V. Ramzaev, 8 Mira Str., Saint-Petersburg, 197101, Russia; Institute of Radiobiology of the National Academy of Sciences of Belarus, 4 Fedyuninskogo Str., Gomel, Belarus

## Abstract

*In situ* gamma-spectrometric measurements were performed at grasslands (45 plots) and forests (6 plots) in the vicinity of the Belarusian nuclear power plant in September–October 2019. The aim of the study was to evaluate the baseline level of ambient dose equivalent rates of gamma radiation from natural radionuclides and ^137^Cs in the period preceding the commissioning of the NPP. The study revealed more than a 2-fold variability in values of the total ambient dose equivalent rate: from 29 to 72 nSv/h. This spread can be explained by variability in the content of natural radionuclides in the environment and, accordingly, ambient dose equivalent rate. At forest sites, compared to grassland sites, the values of ambient dose equivalent rates of gamma radiation from natural radionuclides were statistically significantly lower. The contribution of gamma radiation from ^137^Cs to the total ambient dose equivalent rate was insignificant and averaged 3% for grasslands and 6% for forests.

## Introduction

The first unit of the Belarusian nuclear power plant (BelNPP), located in the Astrovets district (the Grodno region, the Republic of Belarus), was put into commercial operation on 10 June 2021; the second power unit was connected to the network in July 2023^(1, 2)^. Like any other enterprise of the nuclear fuel cycle, the BelNPP is a potential source of radioactive contamination of the environment and, therefore, the state structures of the Republic of Belarus (Ministry of Natural Resources and Environmental Protection represented by Belhydromet) have been monitoring the radiological conditions in the observation zone (radius is 12.9 km) and beyond its borders since 2016^(3, 4)^. Based on the results of determining the content of radionuclides in environmental media and measuring dose rate of gamma radiation in the air, it was concluded that the studied parameters of the radiation environment in the pre start-up period corresponded to the long-term background values typical for this region of the Republic of Belarus. It should be noted that as a result of the Chernobyl accident, this region was not subjected to any significant radioactive contamination: the total density of soil contamination with ^137^Cs from global and Chernobyl radioactive fallout does not exceed 2 kBq/m^2^^(5)^.

The availability of field data on the state of the radiation environment in the pre start-up period around nuclear power plants is a necessary basis for assessing the ‘background’ (‘baseline’) exposure doses of the population living near such facilities (e.g. Harris^(6)^). The data can be used for future re-assessments to verify the stability in the exposure over the time of a NPP operation.

When assessing radiological conditions around nuclear facilities under construction or operation, it is important to have the reliable information not only from governmental authorities but from independent sources as well^(7–9)^. Independent assessments can help to establish mutual understanding and transparency between the facility operator and the authorities on the one hand and the local population on the other hand. At the same time, the observation points of an independent monitoring programme may not completely coincide geographically with the officially selected points. A reasonable generalisation of data from different monitoring systems can give a more representative picture of the radiological conditions that are developing around a radiation hazardous facility.

In 2019, the Lund University (Sweden) initiated an independent research (monitoring) programme to study some key characteristics of the radiation environment (gamma-dose rate in air, activity concentrations (ACs) of radionuclides in soil, water and in various foodstuffs) outside the BelNPP industrial site within the 30-km zone, including the observation zone^(10)^. The Institute of Radiobiology of the National Academy of Sciences of Belarus (Gomel, the Republic of Belarus) and the St. Petersburg Research Institute of Radiation Hygiene named after Professor P.V. Ramzaev (St. Petersburg, the Russian Federation) participated in the programme.

The purpose of this part of the monitoring programme was to separately determine the dose rates of gamma radiation in the air from natural radionuclides and ^137^Cs in outdoor locations.

## Materials and methods

**Figure 1:**
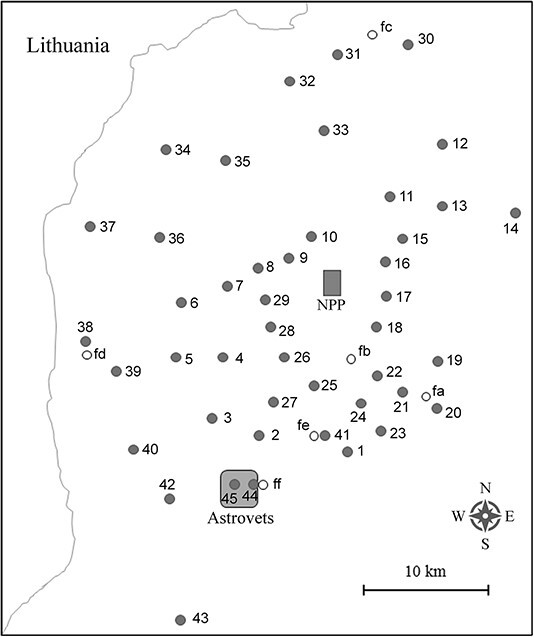
Location of the surveyed grassland plots (filled circles) and forest plots (open circles) in the vicinity of the Belarusian NPP. Geographic coordinates for each plot are provided in Table 1.

The survey was performed in September–October 2019 in the areas of 44 settlements located in different directions from the BelNPP. The location of dosimetric and gamma spectrometric measurement sites in grasslands (45 sites) and forests (6 sites) is shown in Figure 1. The names of the settlements and geographical coordinates of the surveyed plots are provided in Table 1. The distance between the surveyed points and the NPP reactor site varied from 4 to 29 km for grasslands and from 6 to 20 km for forests. The vast majority of the grassland plots was located inside the settlements within the public territory (parks, squares, yards, places for recreation and sports) and in abandoned vegetable gardens (kitchen gardens). All these land plots had been under the influence of human habitation in one way or another for a long time. Such sites can hardly be considered virgin lands in relation to past global and Chernobyl radioactive fallout. The situation is different for the surveyed forest areas: the age of the forests (five pine and one spruce stands) was ˃40 y. Therefore, at least in relation to Chernobyl fallout, the soil in the forests probably corresponded to the category of virgin lands.

**Table 1:** Ambient dose equivalent rate (ADER_tot_), activity concentration of natural radionuclides (NRN) ^226^Ra, ^232^Th, ^40^K and the effective activity concentration (A_eff_) of NRN in soil at individual grassland and forest plots surveyed in the area of the Belarusian NPP in September and October 2019.

Code of plot	Settlement	Geographic coordinates		ADER_tot_ (nSv/h)^b^	Activity concentration (Bq/kg)^c^			
		Latitude°	Longitude°		^226^Ra	^232^Th	^40^K	A_eff_
Grassland								
1	Sosny	54.63655	26.10051	40.6	11.5 (30)	14.0 (17)	403 (6)	64.1 (12)
2	Mali	54.65176	25.98813	56.4	15.4 (25)	25.5 (10)	509 (4)	92.1 (10)
3	Edoklani	54.66488	25.93396	58.0	15.0 (22)	24.7 (10)	549 (4)	93.2 (10)
4^a^	Karveli	54.70678	25.95086	69.6	26.9 (20)	27.4 (13)	591 (5)	113 (11)
5	Lynkishki	54.70784	25.88936	52.1	14.6 (25)	22.9 (11)	440 (5)	82.0 (10)
6	Trokeniki	54.74644	25.90078	44.4	12.2 (25)	19.5 (11)	396 (5)	71.4 (10)
7	Vorona	54.75603	25.95836	71.6	18.4 (22)	32.6 (9)	663 (4)	117 (8.6)
8	Berezovka	54.76996	26.00010	65.3	16.7 (17)	27.6 (7)	613 (3)	105 (9.4)
9	Goza	54.77633	26.03576	49.0	12.1 (30)	20.5 (13)	458 (5)	77.8 (11)
10	Meshlyany	54.78997	26.06812	54.0	14.3 (30)	24.0 (13)	515 (5)	89.5 (11)
11	Mihalishki	54.81542	26.16701	41.7	11.4 (33)	15.7 (16)	406 (6)	66.5 (12)
12^a^	Sidorishki	54.85053	26.23689	39.6	19.3 (21)	11.2 (21)	347 (7)	63.6 (12)
13^a^	Olhovka	54.80737	26.23356	45.7	32.6 (14)	15.8 (16)	292 (8)	78.1 (10)
14^a^	Supronenty	54.79913	26.32256	54.3	25.4 (14)	20.4 (14)	438 (6)	89.3 (10)
15	Markuny	54.78611	26.17885	48.9	14.8 (29)	18.6 (16)	444 (6)	76.9 (12)
16	Skerdimy	54.77054	26.15885	50.7	11.3 (35)	20.2 (14)	485 (5)	79.0 (12)
17	Chehi	54.74374	26.15709	63.8	20.8 (24)	26.0 (13)	576 (5)	104 (11)
18	Gaygoli	54.72216	26.14047	61.8	20.3 (21)	25.3 (11)	578 (4)	103 (10)
19	Gelyuny	54.69830	26.21838	51.5	12.6 (37)	23.8 (14)	478 (6)	84.4 (12)
20	Rymdyuny	54.66327	26.21534	43.0	9.1 (38)	17.6 (14)	422 (5)	68.0 (12)
21	Gudeniki	54.67794	26.17460	43.7	13.0 (35)	18.3 (17)	404 (7)	71.2 (13)
22	Gervyaty	54.69025	26.13979	55.4	14.4 (32)	22.1 (15)	544 (5)	89.6 (12)
23	Galchuny	54.65086	26.14363	48.6	13.3 (34)	19.4 (16)	448 (6)	76.9 (12)
24	Matski	54.67106	26.11876	53.2	14.0 (36)	24.8 (14)	470 (6)	86.5 (12)
25	Chernishki	54.68447	26.06141	53.0	15.4 (35)	22.4 (15)	510 (6)	88.2 (12)
26^a^	Kernyany	54.70431	26.02659	62.6	31.7 (17)	20.9 (15)	492 (6)	101 (10)
27^a^	Filipany	54.67585	26.01169	59.1	27.9 (19)	20.3 (16)	553 (6)	101 (11)
28	Vornyany	54.72754	26.01211	63.9	19.9 (24)	27.8 (12)	589 (5)	106 (10)
29	Bolniki	54.74726	26.00857	62.5	19.3 (27)	26.5 (13)	577 (5)	103 (11)
30	Troschany	54.92423	26.19899	65.6	17.8 (21)	31.1 (9)	597 (4)	109 (9)
31	Barani	54.91928	26.11139	32.1	8.6 (23)	10.4 (13)	284 (4)	46.4 (11)
32	Gadiluny	54.90059	26.04973	40.3	11.0 (31)	14.8 (16)	354 (6)	60.6 (12)
33	Podolcy	54.86507	26.08713	53.5	15.7 (24)	21.7 (12)	491 (5)	85.9 (10)
34	Kemelishki	54.85651	25.89095	46.5	11.1 (29)	17.8 (13)	430 (5)	71.0 (11)
35	Rytan	54.84737	25.96423	51.3	11.3 (32)	21.5 (12)	477 (5)	79.9 (11)
36	Bystrisa	54.79472	25.87961	40.4	14.6 (21)	13.6 (15)	322 (6)	59.9 (11)
37	Zharneli	54.80615	25.79220	57.4	22.2 (14)	22.8 (9)	491 (4)	93.8 (9)
38	Osinovka	54.72300	25.78039	62.1	22.3 (15)	24.5 (9)	537 (4)	100 (9)
39	Zakharishki	54.70183	25.8169	53.5	17.2 (21)	24.6 (10)	417 (5)	84.9 (9)
40	Dreveniki	54.64529	25.83516	55.1	15.4 (21)	23.4 (9)	504 (4)	88.9 (9)
41	Radjuli	54.64879	26.07418	49.6	15.5 (20)	19.2 (11)	412 (4)	75.7 (10)
42	Palushi	54.60683	25.87496	47.0	11.7 (28)	20.9 (11)	420 (5)	74.7 (10)
43	Grodi	54.52358	25.88515	61.7	16.9 (21)	28.1 (9)	542 (4)	99.8 (9)
44	Astrovets	54.61570	25.98162	61.6	15.3 (31)	28.3 (12)	602 (5)	104 (11)
45	Astrovets	54.61631	25.95807	58.1	15.4 (22)	26.7 (9)	513 (4)	94.0 (9)
Forest								
fa	Rymdyuny	54.67538	26.20084	36.6	10.9 (37)	14.2 (20)	304 (8)	55.3 (14)
fb	Gervyaty	54.70116	26.10676	48.7	11.4 (41)	21.9 (15)	373 (7)	71.7 (12)
fc	Barani	54.93326	26.15801	28.6	5.1 (53)	6.7 (20)	237 (7)	37.9 (14)
fd	Osinovka	54.71887	25.78082	48.7	17.8 (22)	18.2 (14)	406 (6)	76.2 (11)
fe	Radjuli	54.64952	26.06282	51.6	16.9 (23)	23.8 (11)	353 (6)	78.1 (10)
ff	Astrovets	54.61599	25.98410	51.7	10.8 (36)	21.8 (13)	459 (5)	78.4 (10)

^a^The survey of the plot was done during rain.

^b^Statistical uncertainty of a measurement does not exceed 2% (95% probability).

^c^Statistical uncertainty in % (95% probability) of a measurement is shown in brackets.

The expeditionary surveys of the territory around the BelNPP had time limits, so the measurements were carried out regardless of weather conditions. Staying in all forest areas and 39 meadow areas fell on periods of dry weather. In six meadow plots, surveys were carried out during rain. The rainy weather did not prevent sampling of environmental media but could affect results of gamma spectrometric and dosimetric measurements due to the deposition of radioactive daughter products of radon decay (RDP), ^214^Bi and ^214^Pb, from the atmosphere onto the earth’s surface with water drops^(11, 12)^. In this regard, six meadow plots surveyed in rainy weather (indicated by the letter ‘a’ in Table 1) were assigned to a separate group.

**Figure 2:**
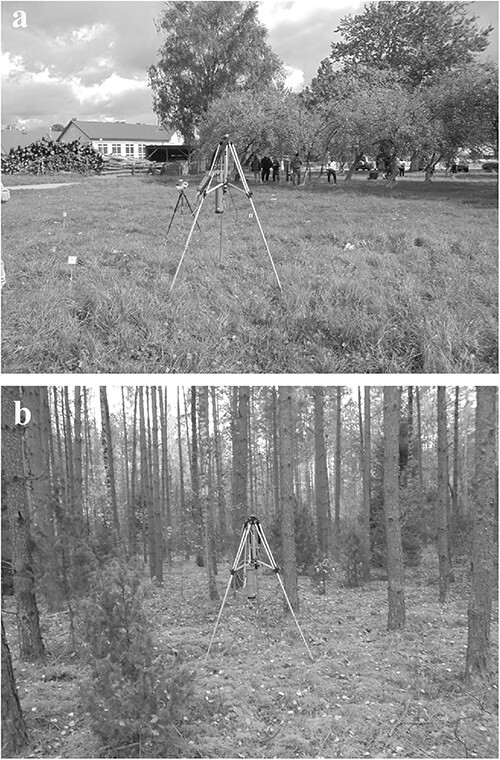
Placement of MKS AT6101D at the grassland plot No. 7 in Vorona (**a**) and at the forest plot ‘fe’ near Radjuli (**b**).

In the sites taken for the survey, an open (in case of the ‘grassland’ location) and flat plot was selected, and in its centre an area with dimensions of 4.5 × 4.5 m^2^ was chosen for the different assessments. Measurements of field (*in situ*) gamma spectra and dose rates of gamma radiation were carried out in the centre of the plot. The portable gamma spectrometer-dosemeter MKS AT6101D (ATOMTEX, the Republic of Belarus) was used for the measurements. The device was placed on an aluminium tripod so that the distance between the NaI(Tl) detector crystal and the ground surface was 1 m (Figure 2). The duration of spectrum acquisition was in the range from 600 to 2180 s (average = 1020 s).

For energy calibration of the spectrometer-dosemeter, the manufacturer (ATOMTEX) used reference point sources of gamma radiation (^22^Na, ^54^Mn, ^57^Co, ^60^Co, ^88^Y, ^109^Cd, ^113^Sn, ^139^Ce, ^137^Cs, ^152^Eu, ^228^Th and ^241^Am). The device was calibrated by the manufacturer to measure total ambient dose equivalent rate of gamma radiation in air (ADER_tot_, nSv/h). The spectrum-to-dose conversion functions were used to convert the measured pulse height distribution into the operational quantity of ambient dose equivalent. The validity of the spectrum-to-dose conversion functions was checked by the manufacturer in a series of measurements using a strong standard ^137^Cs source. The measured doses agreed well (within ±3%) with the calculated doses. However, the maximum basic relative error of measurement of ADER_tot_ at the wide energy range of 50–3000 keV was set by the manufacturer at a level of ±20%. The intrinsic noise of the detector and its response to cosmic radiation is rather small: totally, 8 nSv/h at the sea level^(13)^. This value and the maximum level of basic relative error of the ADER_tot_ measurement should be taken into account when comparing results of the gamma-dose rate measurements obtained by the AT6101D device and by other dosemeters, which may differ from AT6101D in response to gamma radiation and in the intrinsic noise value.

The device MKS AT6101D has the option to measure AC (Bq/kg) of natural radionuclides ^40^K, ^226^Ra and ^232^Th in soil. Large volume activity measures of ^40^K, ^226^Ra and ^232^Th were used by the manufacturer to calibrate the spectrometer to determine ACs of these radionuclides in soil. Basic relative error of measurement of AC of ^40^K, ^226^Ra and ^232^Th does not exceed ±20%. In addition, the programme built into the device makes it possible to calculate the effective AC of natural radionuclides in soil (A_eff_, Bq/kg) and the density of soil contamination with ^137^Cs (kBq/m^2^) (www.atomtex.com). More detailed information on the calibration of the gamma spectrometer and the validation of the measurement results can be found in the paper by Ramzaev *et al*.^(13)^

In addition to the options provided by the manufacturer, Ramzaev *et al*.^(14, 15)^ calibrated the AT6101D device to separately determine values of the ambient dose equivalent rate from natural radionuclides (ADER_NRN_, nSv/h) and ^137^Cs (ADER_Cs_, nSv/h). We used both the options offered by the manufacturer of AT6101D and the methods described in the study of Ramzaev *et al*.^(14)^ The discriminative determination of the natural and technogenic components of the gamma radiation dose rate is necessary for further monitoring studies in this region using gamma spectrometers. In addition, the availability of regional data on the natural component makes it possible to correctly estimate its contribution to the total dose rate of gamma radiation in the air, if measurements are performed using routine dosemeters in the event of a radiation accident.

Statistical processing of the obtained data was carried out using EXCEL for Windows and an on-line free access platform (https://www.socscistatistics.com).

## Results and discussion

**Figure 3:**
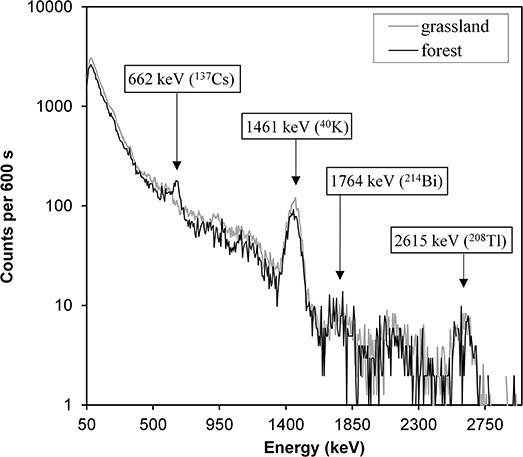
Gamma-ray spectra measured at grassland and forest plots in the area of Rymdyuny.

Figure 3 shows an example (the settlement Rymdyuny) of gamma-ray spectra measured in grasslands and forests in good weather. The spectrogram shows photo peaks of the following natural radionuclides: ^40^K (1461 keV), ^214^Bi (1764 keV) from the ^226^Ra family, and ^208^Tl (2615 keV) from the ^232^Th family. The 662 keV photopeak associated with ^137m^Ba (the short-lived daughter product of ^137^Cs decay) was not visually detected in the spectra from the grasslands. At the same time, a weak photopeak with the energy of 662 keV could be identified on the spectrograms in all the forests surveyed. The presence of this photopeak in the spectra is obviously associated with the presence in the environment of residual amounts of the ^137^Cs radionuclide that deposited to the ground from the atmosphere after nuclear weapons tests and the Chernobyl accident in the past century. On the used and ploughed grassland sites, this radionuclide should be present as well, but at a greater depth in the soil. Under these conditions, at the same value of the radionuclide inventory in the soil, the counting rate in the 662 keV peak at grassland plots will be significantly lower compared to that at forest plots (e.g. Ramzaev *et al*.^(16)^). With the energy resolution of our NaI(Tl) detector of 8% (662 keV), the 662 keV peak itself, due to its very small area, can hardly be distinguished against the background contribution of radiation from natural radionuclides (see also Figure 2 in Ramzaev *et al*.^(14)^). It will be possible to confirm or reject the presence of ^137^Cs in soil of the grassland areas based on results of laboratory analysis of samples taken near the BelNPP^(10)^.

**Figure 4:**
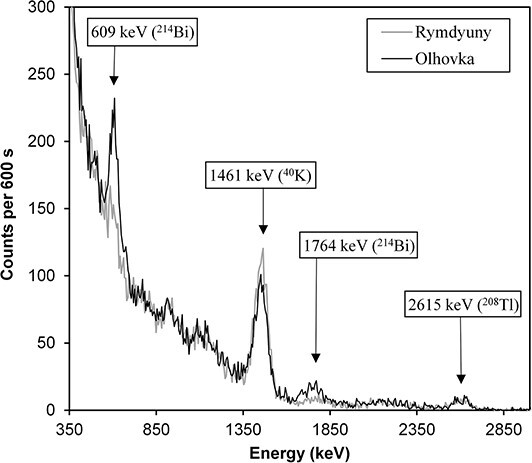
Gamma-ray spectra measured at grassland plots during period of dry weather (Rymdyuny) and during a rain event (Olhovka).

On the gamma spectra measured during rain, compared to the spectra measured in dry weather (see an example in Figure 4), the peaks with the energy of gamma rays of 609 and 1764 keV from ^214^Bi are seen more clearly, which could be expected based on results of other authors^(11, 12)^.

Results of the measurement of ADER_tot_ and AC of natural radionuclides for each of the sites are presented in Table 1. Table 2 contains statistical summary of results of the determination of ADER_tot_ and AC of natural radionuclides, A_eff_, ADER_NRN_ and ADER_Cs_ by groups.

**Table 2:** Summary statistics of the activity concentration of natural radionuclides (NRN) ^226^Ra, ^232^Th, ^40^K and their ratios, the effective activity concentration (A_eff_) of NRN in soil, total ambient dose equivalent rate (ADER_tot_), ambient dose equivalent rate from NRN (ADER_NRN_) and ambient dose equivalent rate from ^137^Cs (ADER_Cs_) for the groups of grassland and forest plots surveyed in the area of the Belarusian NPP in September and October 2019.

Parameter	AC (Bq/kg)			AC ratio			A_eff_ (Bq/kg)	ADER_tot_ (nSv/h)	ADER_NRN_	ADER_Cs_ (nSv/h)
	^226^Ra	^232^Th	^40^K	^232^Th/ ^226^Ra	^40^K/ ^226^Ra	^40^K/ ^232^Th				
Grassland total (*n* = 45)										
Minimum	8.6	10.4	284	0.48	9.0	17.0	46.4	32.1	23.7	−2.1
Maximum	32.6	32.6	663	1.94	46.4	31.0	117	71.6	59.7	4.0
Median	15.4	22.1	485	1.45	32.7	22.1	86.5	53.5	44.1	1.7
Mean	16.6	21.9	480	1.41	31.0	22.3	86.0	53.3	43.9	1.5
S.d.	5.6	5.0	88	0.37	7.7	2.7	15.9	8.9	8.1	1.3
CV, %	34	23	18	26	25	12	18	17	18	87
Grassland, dry weather (*n* = 39)										
Minimum	8.6	10.4	284	0.93	22.1	17.0	46.4	32.1	23.7	−0.6
Maximum	22.3	32.6	663	1.94	46.4	28.8	117	71.6	59.7	3.9
Median	14.8	22.8	485	1.53	33.1	22.1	85.9	53.2	43.8	1.7
Mean	14.9	22.3	484	1.52	33.2	22.1	85.2	53.0	43.5	1.6
S.d.	3.4	4.9	84	0.25	5.4	2.3	15.7	8.6	8.0	1.1
CV, %	23	22	17	16	16	10	18	16	18	69
Grassland, during rain (*n* = 6)										
Minimum	19.3	11.2	292	0.48	9.0	18.5	63.6	39.6	32.4	−2.1
Maximum	32.6	27.4	591	1.02	22.0	31.0	113	69.6	57.6	4.0
Median	27.4	20.4	465	0.69	17.6	22.6	95.2	56.7	48.5	0.2
Mean	27.3	19.3	452	0.71	16.9	23.9	91.0	55.2	46.4	0.7
S.d.	4.8	5.4	117	0.19	4.5	4.5	17.9	11.1	9.1	2.4
CV, %	18	28	26	27	27	19	20	20	20	343
Forest, dry weather (*n* = 6)										
Minimum	5.1	9.7	237	1.02	20.9	14.8	37.9	28.6	19.3	0.4
Maximum	17.8	23.8	459	2.02	46.7	24.4	78.4	51.7	40.0	4.1
Median	11.2	20.0	363	1.66	30.3	21.2	74.0	48.7	37.7	2.8
Mean	12.1	18.3	355	1.60	32.2	20.2	66.3	44.3	33.8	2.5
S.d.	4.7	5.4	78	0.41	10.5	3.6	16.4	9.5	8.4	1.6
CV, %	39	30	22	26	33	18	25	21	25	64

S.d. – standard deviation; CV – coefficient of variation.

For the entire sample of surveyed sites (*n* = 51), the measured ADER_tot_ values ranged from 29 to 72 nSv/h (mean = 52 nSv/h). The statistical uncertainty of the individual measurements was ˂2% (95% probability). The results obtained by us do not contradict the results of other radiological studies performed in the area of the BelNPP in 2019. In particular, according to the data of the State Enterprise ‘Belarusian NPP’, the dose rates of gamma radiation at observation points were in the limits of 100–130 nSv/h^(17)^. According to an independent international group of researchers^(18, 19)^, the results of dose rate measurements were in the range of 48–85 nSv/h.

The ADER_NRN_ in the grassland sites surveyed in dry weather (*n* = 39), compared to that in the forests (*n* = 6) surveyed also in dry weather, was statistically significantly (the Mann–Whitney U-test, *P* < 0.05) higher (Table 2). The ADER_NRN_ in the grassland sites surveyed in dry weather was slightly lower than that for the grasslands surveyed in rainy weather (Table 2), but the difference was not statistically significant (the Mann–Whitney U-test, *P* > 0.05). No statistically significant differences were found between two groups of grassland plots in terms of AC of ^40^K and ^232^Th, as well as A_eff_. At the same time, the mean and median values of the ^226^Ra AC at the plots surveyed in the rain were almost two times higher than those for the grasslands surveyed in dry weather. The difference was statistically significant (*P* < 0.01). These quantitative estimates agree with the results of visual analysis of the size of the peaks with energies of 609 and 1764 keV (see above). It should be noted here that use of the concept of ‘activity concentration of ^226^Ra’ in relation to the situation of measuring field gamma spectra during rain is very conditional because only a part of the short-lived RDP has a strict quantitative correlation with the content of ^226^Ra in the soil directly in the surveyed area. The rest of the RDP is washed out by rain from the vast air masses that can form at considerable distances from the measurement site^(20)^.

Due to the wet deposition of RDP during rainfall, the ratios of the ^232^Th AC to the ^226^Ra AC and the ^40^K AC to the ^226^Ra AC in the areas surveyed in the rain were statistically significantly (*P* < 0.01) lower compared to those for the grasslands surveyed in the dry weather (Table 2). In terms of the ratio of the ^232^Th AC to the ^40^K AC, the two groups of sites did not differ. These assessments are consistent with the study of Yakovlev *et al*.^(12)^ where it was found from *in situ* measurements that the rain did not affect the pulse count rate in the energy channels adjusted to detect radiation from K and Th, but it led to a very significant increase in the pulse count rate in the channels for total uranium. Yoshida *et al*.^(20)^ found from *in situ* measurements that rain did not affect the dose rate of gamma radiation from ^40^K (window 1340–1600 keV) and radionuclides of the ^232^Th family (window 2310–3000 keV: ^208^Tl). At the same time, the dose rate from radionuclides of the uranium family (window 1610–2300 keV: ^214^Bi) increased in rainy weather.

The AC values of natural radionuclides in soil in the grassland plots surveyed in dry weather (*n* = 39) were statistically significantly (Spearman’s test, *P* < 0.05) correlated with each other. The value of the correlation coefficient was 0.684, 0.732 and 0.882 for the pair ^226^Ra–^40^K, ^226^Ra –^232^Th and ^40^K–^232^Th, respectively.

The calculated values of ADER_Cs_ at the 45 grassland plots ranged from −2.1 to 4.0 nSv/h (mean = 1.5 nSv/h; median = 1.7 nSv/h). The presence of values with a negative sign is explained by the influence of the uncertainty in calculating ADER_Cs_ as the difference between ADER_tot_ and ADER_NRN_^(14, 21)^. The average statistical uncertainty for calculating ADER_NRN_ was 9% or 4 nSv/h (95% probability). In the forested areas, the calculated ADER_Cs_ values were in all cases higher than 0: range 0.4–4.1 nSv/h (mean = 2.5 nSv/h; median = 2.8 nSv/h). However, there was no statistically significant difference between the grassland and forest sites in terms of this indicator (the Mann–Whitney U-test, *P* > 0.05). The negligibly small mean and median values of ADER_Cs_ were in agreement with the results of *in situ* measurements of the soil contamination density with ^137^Cs: at all plots, the value of the indicator was ˂4 kBq/m^2^ (the lower detection limit for the MKS AT6101D instrument).

Although authors did collect soil samples at the surveyed grassland plots and performed gamma-spectrometry measurement of each sample *ex situ*, this data was not included in the current study. The results of laboratory measurements and comparison between the *ex situ* and *in situ* data will be presented in subsequent papers.

## Conclusion

The field study, which was performed using a portable gamma spectrometer-dosemeter in the region of the BelNPP location during the pre-commissioning period in 2019, revealed more than a 2-fold spread in ADER_tot_ values (from 29 to 72 nSv/h) outside the industrial site of the NPP. This spread was mainly due to variability in the content of natural radionuclides in the environment and, accordingly, ADER_NRN_. The values of ADER_NRN_ were statistically significantly lower at forest plots compared to grassland plots. The contribution of gamma radiation from ^137^Cs to the ADER_tot_ was insignificant and averaged 3% for grasslands and 6% for forests. When using the results in the future, it should be taken into account that a part of the grassland plots (six out of 45) was surveyed in rainy weather.
